# Relationships between Pre- and Postcopulatory Sexually Selected Traits in Green Frogs (*Lithobates clamitans*)

**DOI:** 10.1093/iob/obaf040

**Published:** 2025-11-06

**Authors:** M O Girard, C J Clark, A F Kahrl

**Affiliations:** Department of Biology, Hamilton College, 198 College Hill Rd. Clinton, NY 13323, USA; Department of Biology, Hamilton College, 198 College Hill Rd. Clinton, NY 13323, USA; Department of Biology, Hamilton College, 198 College Hill Rd. Clinton, NY 13323, USA

## Abstract

Sexual selection is thought to be a primary driver of trait evolution. The expression of traits that improve mate acquisition or fertilization success may be linked in organisms that experience intense sexual selection as they must invest sufficient energy into many different sexual traits in order to reproduce. Two prevailing models—the phenotype-linked fertility hypothesis and the trade-off hypothesis—describe potential positive or negative correlations, respectively, between pre- and postcopulatory traits. We examined the interaction between precopulatory traits (belly coloration and forearm size) and postcopulatory traits (sperm morphology, sperm velocity, and sperm count) in male Green Frogs (*Lithobates clamitans).* We found multiple positive and negative correlations between pre- and postcopulatory sexual traits. Interestingly, belly saturation and hue, which are precopulatory traits, were negatively correlated with sperm count and sperm length, suggesting that the expression of these traits is constrained by energetic resources, fitting the trade-off hypothesis. However, male forearm size was positively correlated with both sperm count and sperm velocity but negatively correlated with sperm length. These results suggest that males must make complex investments in suites of sexual traits to maximize fitness in the face of energetic trade-offs.

## Spanish

Se ha aceptado la selección sexual como el conductor principal de evolución de rasgos. La expresión de rasgos que mejoran la adquisición de pareja o el éxito de fertilización pueden ser relacionados en organismos que tienen experiencias intensas en selección sexual como ellos deben invertir suficiente energía hacia muchos diferentes rasgos sexuales para poder reproducir. Hay dos modelos predominantes—hipótesis de fertilidad ligada al fenotipo y la hipótesis de la compensación—que describen correlaciones potencialmente positivas o negativas, respectivamente, entre pre- y postcopulatorio rasgos. Nosotros examinamos la interacción entre precopulatorio rasgos (la coloración de panza y la medida de antebrazo) y postcopulatorio rasgos (la morfología del espermas, la velocidad de espermas, y cantidad de espermas) en los machos de Rana Verde (*Lithobates clamitans*). Nosotros encontramos muchas correlaciones positivas y negativas entre pre- y poscopulatorio rasgos sexuales. Interesantemente, la saturación y el color de panza, rasgos precopulatorio, fueron correlacionados negativamente con la cantidad y lo largo de espermas, sugiriendo que la expresión de estos rasgos son constreñidos por recursos energéticos, esto va de acuerdo con la hipótesis de la compensación. Sin embargo, la medida de antebrazo en machos fue correlacionado positivamente con cantidad y velocidad de espermas, pero negativamente correlacionado con lo largo de espermas. Estos resultados sugieren que los machos deben de hacer inversiones complejas en conjuntos de rasgos sexuales para maximizar la aptitud cuando balanceando la compensación de energía.

## Introduction

Sexual selection is considered a main driver of phenotypic evolution. It is often divided into two sequential phases: precopulatory selection and postcopulatory selection (Andersson and Simmons 2006). Precopulatory selection occurs before mating, during courtship or competition, and encompasses both traits involved in intersexual interactions such as display coloration, behavior, or plumage (Kodric-Brown 1989; Gerhardt et al. 1996) and intrasexual competition such as weapons or body size (Lappin and Husak 2005; Emberts et al. 2018). In species where females mate multiply, sexual selection continues after mating in postcopulatory selection. For males, traits such as sperm number, size, and performance are often linked with reproductive success during sperm competition (Klaus et al. 2011; Simmons and Fitzpatrick 2012; Kahrl et al. 2021). An individual’s fitness therefore relies upon their success during both pre- and postcopulatory episodes of selection, requiring proper allocation of finite energetic resources between multiple suites of sexual traits as well as other life history traits to achieve the greatest possible fitness (Klaus et al. 2011; Buzatto et al. 2015). However, how males allocate these resources can be very complex and can vary based on many aspects of their mating system and life history (Lüpold et al. 2014; Somjee et al. 2015; Evans and Garcia-Gonzalez 2016; Lüpold et al. 2017; Simmons et al. 2017).

Sexual traits are critical for reproduction and therefore, energy is likely allocated to the traits which yield the highest marginal fitness benefits (Parker et al. 2013). Traits associated with pre- or postcopulatory selection may therefore be correlated with one another in their expression levels due to correlation in the investment of energetic resources to these traits. The phenotype-linked fertility hypothesis (PLFH) and trade-off hypothesis are two models that explain potential correlations between energetic investment into sexual traits. The trade-off hypothesis posits that organisms face a balancing act in the allocation of energy into reproduction and that maximizing mating success or fertility comes at the expense of the other (Parker 1998). The trade-off hypothesis implies a negative correlation between the investment in precopulatory traits and postcopulatory traits. Alternatively, the PLFH states that these energetically “expensive” traits may be linked with male condition (Sheldon 1994). Therefore, males in good condition can express larger, or more high-quality traits, while males in poor condition will have smaller, lower-quality traits. Among males, this pattern generates a positive correlation among the investment of pre- and postcopulatory traits. According to the PLFH, females choose sexually selected phenotypes such as vibrant coloration or specific calling behaviors because these traits are linked with higher fertility (Hettyey et al. 2009a; Sheldon 1994; Blount et al. 2001; Doyle 2011). Female choice may then drive these traits also to covary genetically over time (Simmons and Kotiaho 2002; Hosken et al. 2008; Evans 2010).

The correlation between pre- and postcopulatory traits has been studied on many levels from within individual species (Doyle 2011; Klaus et al. 2011; Travers et al. 2016; Buzatto et al. 2017; Aguiar et al. 2023), across populations (Parker et al. 2013; Lüpold et al. 2017), and in a comparative framework across species (Lüpold et al. 2014; Dines et al. 2015; Kahrl et al. 2016; Supriya et al. 2018; Valencia-Aguilar et al. 2024). How these traits covary can differ dramatically across a variety of species, from positive to negative covariance, and sometimes there are no significant relationships at all (Mautz et al. 2013). In broader taxonomic studies, the direction of the correlations between sexual traits may be in part dictated by the extent of female monopolization across species, where species with high rates of female monopolization (e.g., low rates of female re-mating) or low female promiscuity, have negative correlations between pre- and postcopulatory traits (Lüpold et al. 2014). In species with high monopolization, the opportunity for postcopulatory selection is constrained by strong precopulatory selection, which means that there is little fitness benefit of producing many high-quality sperm (Parker et al. 2013). In species with low female monopolization, there might be complete scramble competition wherein there is a high rate of re-mating, and heavy male investment into postcopulatory traits has a stronger influence on reproductive success (Byrne et al. 2002). Therefore, it appears that when a single episode of sexual selection dominates (e.g., in female monopolization, or complete scramble competition), traits will have a negative covariance, while when both episodes of selection are important for fitness, traits may have a positive correlation (Simmons et al. 2017).

Though suites of sexually selected traits have been examined across many taxonomic groups, very few studies have examined whether pre- and postcopulatory traits are correlated in frogs (Hettyey et al. 2009a; Doyle 2011; Buzatto et al. 2015), and fewer have incorporated multiple suites of traits in one analysis (Aguiar et al. 2023). Despite having the most diverse reproductive strategies of any terrestrial vertebrates (Vági and Székely 2023), frog sperm biology remains understudied outside of descriptions of morphology and tends to focus on comparisons between taxa (Byrne et al. 2003; Aguiar et al. 2006; Salles et al. 2017). We aim to bridge this gap by evaluating a broad suite of sexual traits in a single species (*Lithobates clamitans*) in order to better identify the selective pressures these frogs may face.

Sexually mature male Green Frogs develop a bright yellow throat and enlarged forearms during the breeding season (Schulte-Hostedde and Schank 2009). Forearm size is a predictor of mating success in Green Frog males, and forearm size and belly color are condition-dependent traits that likely serve as a signal of quality for females (Wells 1978; Schulte-Hostedde and Schank 2009). The male forearms and thumbs are used in intrasexual competition to wrestle for territory, secure amplexus, and monopolize females during oviposition, and they may also serve as a signal of body size (Schulte-Hostedde and Schank 2009; Sherman et al. 2010). Males call from their territories with a high posture which showcases their throat, belly, and forearms as they call to females (Wells 1978). The role of coloration is not known in Green Frogs, but research in other anuran species suggests that brighter male coloration can act as an honest signal to females (Vásquez and Pfennig 2007; Bell et al. 2017). Green Frogs mate in ponds with a high population density where females can lay multiple clutches of eggs throughout the breeding season (Wells 1978; Schulte-Hostedde and Schank 2009), and males attempt to monopolize each instance of mating with amplexus. Typically only a single male partner fertilizes the egg clutch during each reproductive episode suggesting there may be strong precopulatory sexual selection for access to each of those mating opportunities (Wells and Schwartz 2001).

However, postcopulatory traits are still extremely important for Green Frogs due to the challenges of their reproductive environment (Dziminski et al. 2010). Aquatic fertilizers, such as the Green Frog, face strong dilution effects of the environment, which may result in selection for sperm traits that boost motility and sperm count to increase fertilization success (Dziminski et al. 2010; Browne et al. 2015). Green Frogs likely produce significant amounts of sperm to fertilize large clutches of eggs (each numbering in the thousand) repeatedly over the reproductive season (Wells 1978; Emerson 1997; Meshaka and Hughes 2014) potentially incurring a large energetic cost (Olsson et al. 1997). Studies across species of frogs show that sperm competition selects for larger testes (Byrne et al. 2003) and larger sperm (Byrne et al. 2003; Liao et al. 2018). In vitro fertilization studies within species of *Crinia* show that slower sperm, with a high percentage of motile sperm have higher fertilization success (Dzminiski et al. 2010). These studies suggest that sperm size and motility may be important ejaculate traits for other externally fertilizing species of frogs (Byrne et al. 2003).

**Fig. 1:**
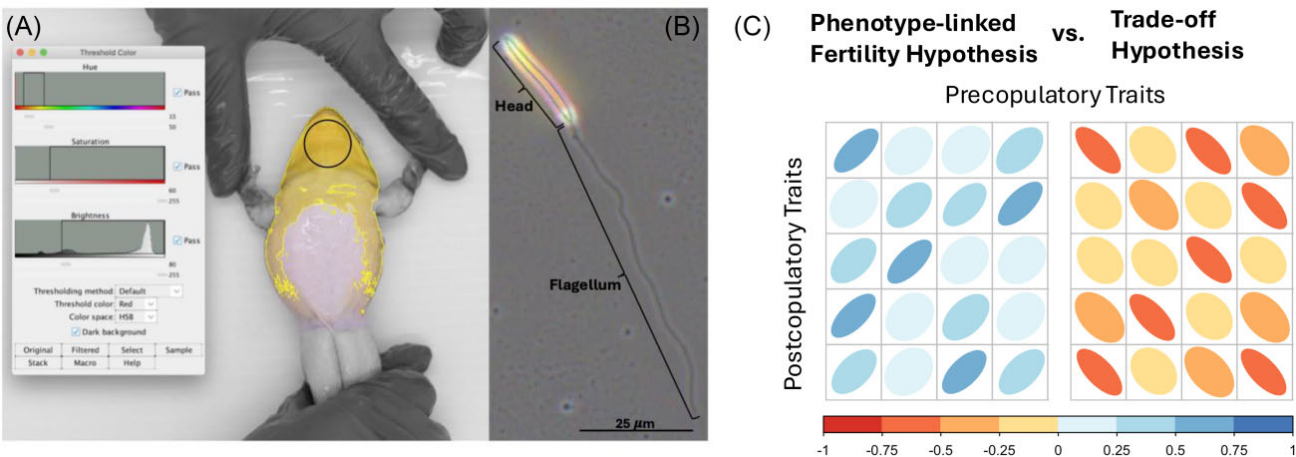
Visualization of several of the traits we measured. (**A**) Methods shown for measuring the saturation and % coverage of the yellow throat. A color threshold in ImageJ includes all yellow coloration within the picture. This is used to calculate the percentage of belly and throat with yellow color. The circle on the throat is the area where hue and saturation were measured for each male. (**B**) *Lithobates clamitans* sperm cell, with head (includes midpiece) and flagellum. (**C**) Predictions of correlation matrices of pre- and postcopulatory traits under the PLFH and the trade-off hypothesis. The shape and color of the ellipses represent the strength of the correlation between the traits, with blue colors indicating positive correlations and red/orange colors indicating negative correlations.

In this study, we test alternative hypotheses, the PLFH (Sheldon 1994) and the trade-off hypothesis (Parker 1998), in male Green Frogs, across a suite of pre- and postcopulatory selected traits. To do this, we focus on throat coloration and forearm size as precopulatory traits as they are involved in signaling and male–male competition (Hettyey et al. 2009a; Schulte-Hostedde and Schank 2009; Doyle 2011) and compare relationships with these traits with postcopulatory traits (sperm count, morphology, and velocity) to test our alternative models. Given that this species appears to have strong female monopolization, we expect to find mostly negative correlations between pre-and postcopulatory traits as found in other taxa (Fig. 1C, Lüpold et al. 2014). This would then support the trade-off hypothesis since uneven energy expenditure into certain traits would demonstrate a higher fitness benefit of one episode of selection over the other (Parker 1998). Alternatively, we predict that observing positive relationships among traits might indicate that males must compete over both episodes of selection, and that these traits are determined in part by male condition (Fig. 1C, Sheldon 1994). However, if we were to find both positive and negative correlations between certain precopulatory and postcopulatory traits, then we might see evidence of a complex pattern of interrelatedness reflecting ideal combinations of traits experiencing selection and the physiological pathways that govern the production of each trait, or alternatively, that some pleiotropic genes under selection influence multiple aspects of an individual’s phenotype.

## Methods

### Field work

Thirty male Green Frogs were collected from two nearby ponds in Central New York, USA: one on Hamilton College campus (43.056, −75.410), and one on Sadaquada Golf Club property (43.099, −75.315) in June of 2023. Collection took place after dark (between 9 PM and 12 AM) during the middle of the breeding season while breeding males were calling. The males were captured using nets, placed in a safe, moist storage container, and transported to Hamilton College. We used a plastic ruler to measure snout-vent length (SVL, nearest 1 mm) for each frog and digital calipers to measure the forearm length (nearest 0.1 mm), forearm width (nearest 0.1 mm), and the width of the widest part of the front thumb (nearest 0.1 mm). Forearm width was measured midway between the elbow and the wrist of the individuals by using calipers to measure width of the widest diameter of the arm. The weight of each frog (nearest 0.25 g) obtained using a hanging scale. Each measurement type was taken by only one of the authors.

### Belly color imaging

To photograph the frogs, we built a box out of white corrugated plastic with a pair of full-spectrum LED lights on the ceiling to provide standardized and even lighting. The frogs were placed in the box on their backs with their arms and legs spread so that the entire throat and belly of each frog was visible and parallel to the ground. Photos were taken using a Nikon D610 camera (with a constant set ISO, F stop, and frame rate) mounted approximately 0.5 m above the frog positioned in a hole cut in the top of the box. A color standard with a built-in ruler was placed next to the frog in the final images to confirm identical lighting and imaging conditions which we did not need to adjust in our final analysis. The images were loaded into Adobe Photoshop where we used the magnetic lasso tool to select the outline of the frog and then inverted the background to black and white. Then, using ImageJ (Schneider et al. 2012), we traced the total area of the throat and belly (mm^2^) (excluding arms and legs, Fig. 1A). We then used the adjust threshold tool on the traced area to select only sections of the frog falling between a hue of 15–50, saturation of 60–255, and a brightness of 80–255 to isolate the areas with yellow pigment. From there, we used this binary threshold image to measure the area of the belly (mm^2^) and throat with yellow pigmentation and used this area value divided by the total area of the throat and belly (mm^2^) to calculate the percentage of the belly with yellow pigment. The throat universally had the brightest area of color for all males, and since some males had very little color on their belly, we measured the average hue, saturation, and brightness of a circular area that spanned the width and height of the male’s throat without reaching into the belly area (Fig. 1A). While hue and saturation are uncorrelated, saturation and brightness are highly correlated (*R^2^* = 0.39, *t* = −4.37, *P* < 0.0001), we therefore excluded brightness from our models as saturation is likely more indicative of the amount of pigment present in the skin (Brenes-Soto et al. 2017).

Hue is a more complex variable since positive and negative hue doesn’t cleanly correspond with a “strong” or “weak” expression of traits. However, frogs with high carotenoid diets tend to develop yellow areas with a lower hue (a slight shift towards orange), while frogs without tend to develop higher hue values (shift toward green) on their yellow chests (Umbers et al. 2016; Stuart-Fox et al. 2021). Therefore, because lower hue signifies a more energetically expensive state, we multiplied our hue values by −1 for our models to reverse the direction of our values, while retaining the data’s structure. In this way, a high value of hue would indicate the potential for a high level of carotenoids (more orange) and allow us to see the typical correlational patterns expected for other traits (larger values = more energy).

### Sperm collection and analysis

To obtain sperm samples from the frogs, we used hormonal stimulation using Amphiplex (Trudeau et al. 2010), which induces spawning in amphibians. Each frog was given an injection of Amphiplex proportional to their body weight following Trudaeu et al. (2010), then sperm samples used for analysis were collected at 60 min post-injection by gently applying pressure to the frog’s abdomen just above the pelvis to express spermic urine from the frog onto a petri dish. To standardize our measurement for sperm count, we collected urine samples from every individual 30 min post-injection to clear the bladder and then kept the individuals in identical containers with water so that the males would produce roughly equal volumes of spermic urine. We suspended the sperm sample in deionized water to make a total volume of 1 mL. We immediately transferred a 10 µL of the suspension to a Cell Vision Slide (Cell Vision Technologies, Heerhugowaard, The Netherlands) and used computer-assisted sperm class analyzer software (SCA, Microptic, Barcelona, Spain) to record and analyze sperm motility data. Recordings were made using a positive phase contrast objective at 10× magnification on a Nikon CiL microscope (Nikon Inc, Melville, NY, USA), with a Basler ACE 1300 (Basler Inc., Exton, PA, USA) camera. Multiple 1 s videos were recorded from different areas of the slide to maximize the number of motile cells captured. We modified the SCA settings to eliminate debris from the field by including the following settings (head area: 13–30, head elongation: 25–60) and visually inspecting the field to ensure only sperm were included. Males had an average of 256 cells measured in these tracks. From these tracks, we then averaged the straight-line velocity (VSL) of all cells for each male to use in our analyses. We then used the same sperm sample to quantify sperm count using a hemocytometer Petroff-Hausser Counter (cat. # 3900, Hausser Scientific), which was manually counted to estimate the sperm count for each individual. Finally, we used the remainder of the sperm sample to prepare a slide for morphology analysis by preserving each sample in 4% paraformaldehyde before drying them on a slide. We imaged 10 intact cells for each individual using 40× positive phase microscopy on the same Nikon CiL. We then measured the head (including midpiece), and flagellum length for each cell using the segment tool using ImageJ (Fig. 1B, Schneider et al. 2012). Finally, we combined the lengths of the head and flagellum to produce sperm total length and took the mean of each measurement for each individual based on these 10 cells. As this is the first description of sperm for this species, we also examined the variance within and between males by calculating repeatability using the function “rpt” in the package *rptR* (Stoffel et al. 2017). After sampling was complete, all frogs were returned unharmed to their pond of origin.

### Statistical analyses

All analyses were conducted in R Version 4.2.2 (R Code Team 2022). First, we summarized forearm size using a principal components analysis using the function “prcomp” in the *stats* package (R Core Team 2022) since forearms and the enlarged thumb are used together during both the act of amplexus and male-male competition when the males grapple each other (Wells 1978; Schulte-Hostedde and Schank 2009). We included forearm length, width, and toe width as variables in this PCA, and used the first principal component (PC1), in our further analyses. PC1 explained 59.6% of the variance in traits, and all 3 traits loaded positively onto this PC. PC2 and PC3 explained 22% and 18% of the variance in traits, respectively, but the loadings were not positive across all traits, so these PCs were excluded as they would not be as easily interpreted as “forearm size.” Next, we ln-transformed sperm count to adjust for a left skew in the data (all other variables had normal distributions and were not transformed). As our dataset included a large suite of traits, we opted to use backward stepwise model selection (Fernández Pierna et al. 2009) using the “step” function in the *stats* package (R Code Team 2022) to examine the relationships between pre- and postcopulatory traits. These models begin with an initial “full” linear model that includes a single trait and all predictor variables and removes single predictor variables with the highest *P*-values sequentially until an adequate model with the lowest Akaike information criterion (AIC) score is reached (Akaike 2011). To ensure that we limited the chance of type I error in our analyses (Forstmeier and Schielzeth 2011), we compared our best fit models to the global model and intercept-only models (Supplemental Table 1). Since our predictions of how pre- and postcopulatory traits should interact are not causal in nature, we ran our model twice—once using individual precopulatory traits as independent variables and once using individual postcopulatory traits as independent variables with all postcopulatory traits or all precopulatory traits as dependent variables, respectively. We compared variance inflation factors (VIF) to determine how much the variance of our regression coefficients is caused by correlation between independent variables. However, as sperm traits tend to be correlated with one another, this led to higher VIF scores in models where postcopulatory traits were the independent variables. This issue was not the case with the precopulatory traits, where the highest VIF score was 1.78. Therefore, we focused on the models where postcopulatory traits were the dependent variables for our analyses and interpretation. We checked the model fits by examining the linearity of model residuals, Q–Q plots, and checked for homogeneity of variance. Finally, as the PLFH assumes that traits covary due to links with body condition, we also tested for condition-dependence of all pre- and postcopulatory traits using a linear regression between condition and each trait. Condition was calculated using the residuals from a regression of log mass on log SVL. We also checked for correlations between all traits and both body size and condition using linear regressions.

## Results

**Fig. 2:**
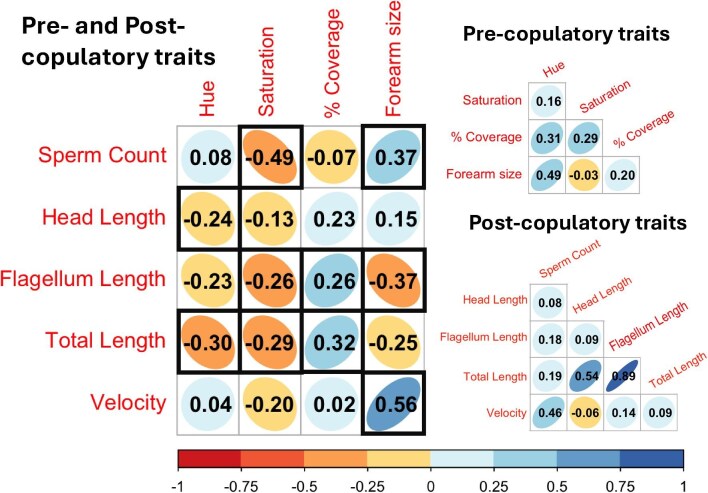
Correlation matrices showing all correlations between precopulatory and postcopulatory traits in male Green Frogs (*Lithobates clamitans*). Correlation values are listed inside of each square, the colors of the correlations indicate positive (blue) or negative (orange) correlations, and the shape of the ellipse represents the structure of the data. Significant positive correlations from our models are surrounded by a bolded square. Correlations within the precopulatory traits and within postcopulatory traits are shown to the right.

We sampled 30 reproductively active males from the field, all of whom produced a sperm sample. Summarized trait values (means, standard deviations) for these males can be found in Table 1. Our analyses found a variety of both positive and negative correlations between precopulatory and postcopulatory traits. Total sperm length and flagellum length had significant negative correlations with belly color saturation, hue, and positive correlations with % belly coverage (Fig. 2, Table 2), likely because the flagellum makes up the majority of the cell length and is highly correlated with total sperm length (as a note, our global model for sperm total length was not significantly different from our “best fit” model, and in the global model sperm, total length was only marginally negatively correlated with hue). Sperm count was also negatively correlated with belly saturation (Table 2). Interestingly, % belly coverage, as well as forearm size had positive correlations with sperm traits (sperm count, sperm velocity, and sperm length [Table 2, Fig. 2]). We found no significant correlations between body condition and any sexual trait except a positive correlation with the hue of throat color, with males in higher condition expressing more orange throats (Supplementary Table 2). As hue is representative of the quantity of carotenoids influencing the coloration of the throats (Umbers et al. 2016), we flipped the direction of our hue variable (see methods), so that higher values would indicate more carotenoids (more orange). We also found that forearm size and % belly coverage and hue were positively correlated with SVL (Supplementary Table 3).

**Table 1:** Trait means ± SD and range of reproductive traits for 30 adult Green Frogs (*Lithobates clamitans*)

Trait	Mean ± SD	Range (Min–Max)
*Physical traits*		
SVL (mm)	74.66 ± 6.39	57–84
Mass (g)	41.44 ± 10.54	19.25–61.25
Precopulatory traits		
Forearm length (mm)	16.46 ± 1.32	14.1–19.1
Forearm width (mm)	8.83 ± 1.48	5.8–11.6
Thumb width (mm)	4.27 ± 0.88	2.5–6.5
% yellow belly	59.11 ± 17.73	26.56–96.04
*Coloration*		
Saturation of belly	52.46 ± 7.08	35.7–64.4
Hue of belly	47.20 ± 2.04	43–52
Postcopulatory traits		
Sperm count	6.349 × 10^7^ ± 9.8 × 10^7^	1.300 × 10^6–^3.8 × 10^8^
Sperm total length (µm)	86.49 ± 3.14	80.98–94.08
Sperm head length (µm)	20.05 ± 1.45	18.35–24.87
Sperm flagellum length (µm)	65.44 ± 2.65	59.77–73.38
Sperm velocity (VSL) (µm/s)	8.99 ± 3.40	2.93–16.35

**Table 2:** *T*-test results run from the selected model of the backward stepwise model selection with the lowest AIC criterion

Trait	*t-*value	*P*-value	*r*²
**Sperm count**			0.324
Saturation	−2.681	**0.012**	
Forearm size (PC1)	2.330	**0.027**	
**Total sperm length**			0.417
Saturation	−2.446	**0.022**	
Hue	−2.634	0.014**	
Percent coverage	3.458	**0.001**	
**Head length**			0.246
Hue	−2.508	**0.018**	
Percent coverage	1.764	0.089	
Forearm size (PC1)	−1.461	0.097	
**Flagellum length**			0.412
Saturation	−2.636	**0.014**	
Percent coverage	2.954	**0.006**	
Forearm size (PC1)	−3.125	**0.004**	
**Sperm velocity**			0.386
Forearm size (PC1)	4.117	**≤0.001**	
Hue	−1.807	0.082	

*Note:* Bolded variables are postcopulatory traits which we used as the independent variables. All precopulatory traits which were retained in the final models are shown below the sperm traits. Significant p-values are bolded.

**= In global model, which was not significantly different from the best fit model, Hue was no longer significant with a *P* = 0.093.

This is the first description of Green Frog sperm morphology to our knowledge. The sperm morphology (Fig. 1B) resembles other *Lithobates* species, having a long oval-shaped head, small midpiece and single flagellum (Kuramoto 1998; Fitzpatrick et al. 2022). However, the total length of the sperm is slightly longer than most other Ranoidea frogs, averaging 86.5 ± 3.14 μm in our sample (Table 1), while most Ranoidea frogs tend to have sperm closer to 50 μm (Kuramoto 1998; Fitzpatrick et al. 2022). Though males do show variation in the size of their sperm components (Supplemental Fig. 1), the repeatability of sperm length in this population is moderate (head length = 0.388 ± 0.07, flagellum length = 0.336 ± 0.06, and total length = 0.374 ± 0.07).

## Discussion

This study is one of only a handful that examines covariance between pre- and postcopulatory traits in frogs (Hettyey et al. 2009a; Doyle 2011; Parker et al. 2013; Buzzatto et al. 2017; Lüpold et al. 2017; Aguiar et al. 2023). It is the only one that includes analyses examining a suite of traits that includes coloration and forearm size as well as a full suite of sperm traits, including sperm velocity. We found both positive and negative correlations between our pre- and postcopulatory traits, demonstrating support for both the PLFH and the trade-off hypothesis (Fig. 2, Table 2, Sheldon 1994; Parker 1998). However, most of the significant correlations we found were negative (6/10), and therefore our findings mostly support our predictions we made where many pre- and postcopulatory traits would be negatively correlated due to high female monopolization in this species (Lüpold et al. 2014). This mosaic of correlations is similar to previous findings in frogs (Doyle 2011; Aguiar et al. 2023), and below we discuss how the life history and physiology of the Green Frog may contribute to this pattern.

We found several negative correlations between traits associated with carotenoid expression (saturation and hue) and sperm traits (sperm count, total length, head length, and flagellum length, Fig. 2, Table 2), perhaps emphasizing the costs of sperm production and the concentration of pigments within chromatophores in this group (Ogilvy et al. 2012; Brenes-Soto et al. 2017). Since carotenoid concentration impacts yellow color saturation and hue in other members of Anura (Umbers et al. 2016), we can use it as a proxy for dietary carotenoid consumption. In this case, the significant negative relationships we found indicate that frogs with fewer and shorter sperm cells tend to have more carotenoids in their diet. Our study found that bellies with an oranger hue were associated with high body condition and larger SVL (Supplemental Tables 2 and 3), but since we did not find any positive correlations between hue and other traits, these relationships do not support the PLFH (Sheldon 1994). Therefore, the trade-off hypothesis seems to be a more appropriate explanation for the phenomenon we observe: male Green Frogs must allocate energy between ejaculate expenditure and the acquisition and incorporation of dietary carotenoids into their ventral throat coloration (Rudh and Qvanström 2013; Umbers et al. 2016; Brenes-Soto et al. 2017).

Our results contrast with patterns found in other species, which mostly find support for the PLFH when examining relationships between color and sperm. In other species, particularly birds and fish, carotenoids can play a role in sperm traits such as increasing sperm density (Rowe et al. 2010), sperm number (Pitcher and Evans 2001), sperm longevity (Fukuda and Karino 2014), and sperm motility (Peters et al. 2004; Janhunen et al. 2009; Beausoleil et al. 2012; Navara et al. 2012), but in some cases trading-off with sperm size (Hasegawa et al. 2022) and sperm performance under oxidative stress (Tomásek et al. 2017). For other anurans, color does not seem to be related sperm traits (Hettyey et al. 2009b), but evidence is very limited, and the only other species examined did not focus on a carotenoid-based color. In general, very few researchers have examined coloration and sperm traits in a natural setting, and the majority of studies found positive correlations with sperm number, velocity, and motility, and very few examined sperm size. Some interpret color as being an “honest signal” of quality or hypothesizing that carotenoids can act as antioxidants protecting sperm from damage from reactive oxygen species (Helfenstein et al. 2010). It may be that our results contrast with these other patterns because of the difference in the colors of the traits, Green Frogs display yellow/orange, while many other species cited above display red coloration, leading to different levels of circulating carotenoids, potentially having an impact on sperm cells only at high levels.

However, we interpret our results in light of the energetic costs and trade-off of the traits we examined in this study. The production of both ejaculates and throat coloration seems to be energetically expensive in Green Frogs, potentially leading to the negative correlations we see between these traits. In anurans, the risk of sperm competition drives increases in both sperm size and sperm number (Byrne et al. 2002, 2003), and sperm number is one of the primary targets of reproductive success in frogs (Dziminski et al. 2010) suggesting that selection may drive allocation of resources to both traits when sperm competition is high (Liao et al. 2018). For Green Frogs, who mate over an extended reproductive season, producing many large ejaculates is likely very energetically costly and may shift allocation of resources away from other traits to ensure reproductive success. For other anurans with long reproductive seasons, a decrease in ejaculate size and fertilization success is observed as the breeding season passes (Hettyey et al. 2009b). Similarly, the production of colorful displays also comes at an energetic cost (Ogilvy et al. 2012; Parker 2016; Brenes-Soto et al. 2017). Green Frogs, like many anurans, change their coloration to a bright yellow during the breeding season, primarily around the throat and belly (Bell et al 2017). The bright throat colors that the males develop are produced by xanthophores, a type of chromatophore which contain high amounts of yellow pigments (Richards and Nace 1983; Rudh and Qvanström 2013; Bell et al. 2017; Alfakih et al. 2022). Though the genetic pathways of pigmentation in Green Frogs are unknown, the primary yellow pigments that this species expresses are pteridines, an endogenous pigment, and carotenoids, an exogenous pigment (Obika and Bagnara 1964; Rudh and Qvanström 2013; Umbers et al. 2016; Alfakih et al. 2022). In particular, the act of incorporating carotenoids is energetically costly to frogs since they do not produce carotenoids on their own but must obtain them from insects they consume (Umbers et al. 2016). As frogs with diets that contain more carotenoids tend to have more saturated yellow pigmentation, and a shift toward orange in the hue in their yellow patches (Umbers et al. 2016; Stuart-Fox et al. 2021), this may indicate that males allocate significant energy into acquiring carotenoid-rich prey and/or expressing higher concentrations of the pigment (Bell et al. 2017; Stuart-Fox et al. 2021; Alfakih et al. 2022).

Though we observed several negative correlations with color and sperm traits, we also observed positive correlations between the % of yellow belly coverage and sperm traits (flagellum length and total length) (Table 2). The % of yellow belly coverage measured the area of the belly with any yellow pigmentation and does not necessarily reflect the concentration or type of pigments present in the skin. Because of this, individuals with a higher % coverage do not necessarily have a high dietary carotenoid content or have a large concentration of pigments in each of the xanthophores that are expressing color (Bell et al. 2017; Brenes-Soto et al. 2017; Alfakih et al. 2022). This means that the physiological cost of % coverage is harder to estimate than it is for hue or saturation and it is not clear if these positive correlations truly provide support for the PLFH (Umbers et al. 2016; Brenes-Soto et al. 2017; Alfakih et al. 2022).

Unexpectedly, we found a mix of negative and positive correlations between forearm size and sperm traits. Sperm count and velocity were positively correlated with forearm size, while flagellum length was negatively correlated with forearm size. The positive correlation between forearm size and both sperm count and sperm velocity could indicate support for the PLFH. Though none of these traits are correlated with condition in our study (but see Schulte-Hostedde and Schank 2009), forearm size is correlated with body size suggesting that either older males or males able to acquire more resources can produce larger forearms (both SVL and mass, Supplemental Table 3). Forearms are used for both holding onto the female during amplexus and for wrestling rival males to establish territories or remove them from females during mating (Wells 1978; Howard and Kluge 1985). The size of the forearm is primarily due to increases in the muscular structure mediated by increases in androgens (Sidor and Blackburn 1998; Peters and Aulner 2000), and males with larger forearms have higher reproductive success in toads (Lee 2001). As forearm size is also a sexually dimorphic trait, the positive correlation between forearm size and sperm traits may be hormonally linked. For example, larger males may produce more androgens, which in turn result in more exaggerated sexually dimorphic features such as forearm size and larger, better performing ejaculates (Lofeu et al. 2017).

The relationship between sperm velocity and forearm size is harder to interpret since selection on other anurans favors slower sperm with a large percentage of motile sperm (Dziminski et al. 2010). Anuran sperm takes a long time to penetrate the jelly of the eggs, and there may be a trade-off between high speed and high endurance in sperm though we do not know if this is the case in Green Frogs (Dziminski et al. 2010; Liao et al. 2018). Our measure of velocity may also reflect sperm motility, in which case males with more androgens make bigger forearms and more sperm with better function. It is not entirely clear what could mediate the negative relationship between forearm size and flagellum length, but perhaps large males who invest in many sperm experience a within ejaculate trade-off, where sperm count is increased at the expense of flagellum length (Parker 1993). However, we do not have a clear understanding of how selection acts on sperm in this species nor whether subtle differences in sperm length between males incurs an energetic cost. One other potential mechanism that could mediate the relationship between forearm size and sperm traits is population density or operational sex ratio (Lüpold et al. 2017). Species of frogs with low density and/or a more female-biased operational sex ratio show a positive correlation between forearm size and testes size (which can be a proxy for sperm count). Additionally, species who are aquatic fertilizers and had single-male amplexus (such as Green Frogs) showed more positive correlations between forearm and testis size (Lüpold et al. 2017). Future studies could focus on disentangling these relationships and finding possible physiological mechanisms or patterns of selection that could explain the divergent patterns between forearm size and sperm traits.

Though our results may provide some evidence to support the PLFH or trade-off hypothesis, further experimental studies are needed to concretely demonstrate that these traits are experiencing energetic trade-offs and/or are linked with reproductive success in this species. While we have strong evidence that these traits are important for other related families of frogs (Gerhardt et al. 1996; Lee 2001; Bell et al. 2017), there has been very little research on the fitness consequences of color and sperm traits in *Lithobates.* Since both of these hypotheses come with the assumption that these traits are linked with reproductive success, we call for future research to investigate the links between reproductive traits, reproductive success, and metabolic processes that may indicate the energetic cost of producing these traits. Additional traits that should be included in future studies are vocalization traits. Green Frogs exhibit several call types for advertisement or territoriality (Wells 1978; Bee and Perrill 1996). Traits such as call effort (meaning calls per minute) or amplitude, might easily translate into an interpretable energetic cost, while other aspects of call such as frequency or the structure of the sound (rise and fall time), might not be as clear. Compared to many species of anurans such as American Toads (*Anaxyrus americanus*) or Gray Tree Frog (*Dryophytes versicolor*) (Zweifel 1968; Wells and Taigen 1986), Green Frogs call frequency is slow (minutes between calls), and short. For this reason, and because they are not well characterized in terms of their energetic cost, we excluded them for this study.

In summary, our findings find evidence supporting primarily the trade-off hypothesis with some support for the PLFH in male Green Frogs. We found negative correlations between pigmentation and sperm production/size, as well as between forearm size and sperm size which supports the trade-off hypothesis since both traits are energetically expensive in many species (Godwin et al. 2017; Alfakih et al. 2022). The positive correlation between forearm size and sperm count may yield some evidence of the PLFH since sperm count is a predictor of fertilization success in frogs (Dziminski et al. 2010) and forearm size is both condition-dependent (Schulte Hostedde and Schank 2009) and linked with male mating success (Greene and Funk 2009). Thus, the males with large forearms may be “accurately advertising” their fertility (Bell et al. 2017; Godwin et al. 2017). We hope that this research can be used in further evaluations of pre- and postcopulatory dynamics and demonstrate the efficacy of comprehensive evaluations of broad suites of precopulatory traits used in studies of sexual selection. Testing these hypotheses can help yield new understandings of core biological principles as we learn the drivers of sexual selection and the way precopulatory and postcopulatory traits interact (Pélissié et al. 2014; Marie-Orleach et al. 2021).

## Supplementary Material

obaf040_Supplemental_Files

## Data Availability

Data are available as a supplemental file.
