# Supplementary material for: Relationships between Pre- and Postcopulatory Sexually Selected Traits in Green Frogs (Lithobates clamitans)
Source: Integr Org Biol. 2025 Nov 6;7(1):obaf040. doi: 10.1093/iob/obaf040 (PMC12628315; doi:10.1093/iob/obaf040)
Supplement: obaf040_Supplemental_Files [file obaf040_supplemental_files.zip › Final 0035_Supplemental Tables and Figure .docx]

**Supplemental Table 1:** Comparisons of the best fit model from backwards step elimination to the global model and intercept-only model using AIC values. Differences between AIC scores are noted with ΔAIC, and Akaike weights (ω_i_).

| Model | AIC | ΔAIC | ω_i_ |
| --- | --- | --- | --- |
| **Sperm Count** |  |  |  |
| Best fit model | 21.41 | 0 | 0.81 |
| Global model | 24.33 | 2.92 | 0.18 |
| Intercept only | 116.30 | 94.89 | 0 |
| **Total Sperm Length** |  |  |  |
| Best fit model | 59.42 | 0 | 0.50 |
| Global model | 59.43 | 0.01 | 0.50 |
| Intercept only | 156.77 | 97.35 | 0 |
| **Head Length** |  |  |  |
| Best fit model | 21.00 | 0 | 0.63 |
| Global model | 22.14 | 1.14 | 0.36 |
| Intercept only | 110.63 | 89.63 | 0 |
| **Flagellum Length** |  |  |  |
| Best fit model | 49.58 | 0 | 0.69 |
| Global model | 51.22 | 1.64 | 0.31 |
| Intercept only | 146.67 | 97.09 | 0 |
| **Sperm Velocity** |  |  |  |
| Best fit model | 71.47 | 0 | 0.83 |
| Global model | 74.73 | 3.26 | 0.16 |
| Intercept only | 164.28 | 92.81 | 0 |

**Supplemental Table 2.** Correlations between pre- and postcopulatory traits and body condition for 30 male Green Frogs.

| Trait | t- value | p-value |
| --- | --- | --- |
| Percent Coverage | 0.109 | 0.277 |
| Saturation | 0.393 | 0.698 |
| Hue | 3.276 | **0.003** |
| Forearm size (PC1) | 1.420 | 0.167 |
| Head Length | 0.301 | 0.766 |
| Flagellum Length | 0.645 | 0.524 |
| Total Length | 0.686 | 0.499 |
| Sperm Count | 0.003 | 0.997 |
| Sperm Velocity | -0.728 | 0.473 |

**Supplemental Table 3.** Correlations between pre- and postcopulatory traits and body size (SVL) for 30 male Green Frogs.

| Trait | t- value | p-value |
| --- | --- | --- |
| Percent Coverage | 2.402 | **0.023** |
| Saturation | 1.225 | 0.231 |
| Hue | 4.307 | **<0.001** |
| Forearm size (PC1) | 4.604 | **<0.001** |
| Head Length | 0.985 | 0.333 |
| Flagellum Length | -0.715 | 0.481 |
| Total Length | -0.150 | 0.882 |
| Sperm Count | 1.076 | 0.291 |
| Sperm Velocity | 0.735 | 0.469 |

**Supplemental Figure 1.** Male means with standard error for sperm head, flagellum and total length for each male in the study.

**Supplemental Figure 1.** Male means with standard error for sperm head, flagellum and total length for each male in the study.
