# Supplementary figures and images for: Relationships between Pre- and Postcopulatory Sexually Selected Traits in Green Frogs (Lithobates clamitans)
Source: Integr Org Biol. 2025 Nov 6;7(1):obaf040. doi: 10.1093/iob/obaf040 (PMC12628315; doi:10.1093/iob/obaf040)

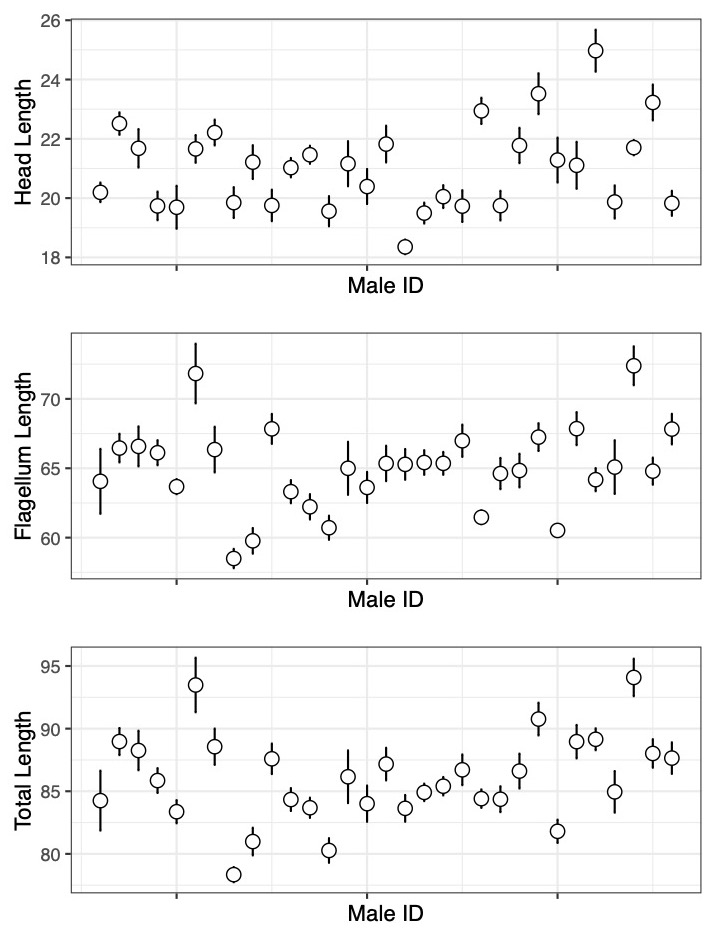

Supplement: obaf040_Supplemental_Files [file obaf040_supplemental_files.zip › Supplemental Figure 1.jpg]
